# Fasting Enhances TRAIL-Mediated Liver Natural Killer Cell Activity via HSP70 Upregulation

**DOI:** 10.1371/journal.pone.0110748

**Published:** 2014-10-30

**Authors:** Vu T. A. Dang, Kazuaki Tanabe, Yuka Tanaka, Noriaki Tokumoto, Toshihiro Misumi, Yoshihiro Saeki, Nobuaki Fujikuni, Hideki Ohdan

**Affiliations:** 1 Department of Gastroenterological and Transplant Surgery, Applied Life Sciences, Institute of Biomedical and Health Sciences, Hiroshima University, Hiroshima, Japan; 2 Department of Surgery, Hiroshima City Hospital, Hiroshima, Japan; The Ohio State University, United States of America

## Abstract

Acute starvation, which is frequently observed in clinical practice, sometimes augments the cytolytic activity of natural killer cells against neoplastic cells. In this study, we investigated the molecular mechanisms underlying the enhancement of natural killer cell function by fasting in mice. The total number of liver resident natural killer cells in a unit weight of liver tissue obtained from C57BL/6J mice did not change after a 3-day fast, while the proportions of tumor necrosis factor–related apoptosis-inducing ligand (TRAIL)^+^ and CD69^+^ natural killer cells were significantly elevated (n = 7, *p* <0.01), as determined by flow cytometric analysis. Furthermore, we found that TRAIL^−^ natural killer cells that were adoptively transferred into Rag-2^−/−^ γ chain^−/−^ mice could convert into TRAIL^+^ natural killer cells in fasted mice at a higher proportion than in fed mice. Liver natural killer cells also showed high TRAIL-mediated antitumor function in response to 3-day fasting. Since these fasted mice highly expressed heat shock protein 70 (n = 7, *p* <0.05) in liver tissues, as determined by western blot, the role of this protein in natural killer cell activation was investigated. Treatment of liver lymphocytes with 50 µg/mL of recombinant heat shock protein 70 led to the upregulation of both TRAIL and CD69 in liver natural killer cells (n = 6, *p* <0.05). In addition, HSP70 neutralization by intraperitoneally injecting an anti- heat shock protein 70 monoclonal antibody into mice prior to fasting led to the downregulation of TRAIL expression (n = 6, *p* <0.05). These findings indicate that acute fasting enhances TRAIL-mediated liver natural killer cell activity against neoplastic cells through upregulation of heat shock protein 70.

## Introduction

Natural killer (NK) cells, the front-line defense for the immune system, do not require priming to exert their effector function on neoplastic cells, modified cells, and invading infectious microbes [1]–[3]. Although it has been demonstrated that acute starvation, which is frequently observed in clinical practice, sometimes augments the cytolytic activity of NK cells against neoplastic cells [4], the molecular mechanisms underlying this phenomenon remain unclear. In addition, few studies have addressed the question of whether such augmentation of NK cell activity by nutritional alteration is of practical benefit.

It has been shown that many transformed cells, including virus-infected and tumor cells, can be attacked by tumor necrosis factor–related apoptosis-inducing ligand (TRAIL)-expressing NK cells [5]–[8]. A variety of mechanisms are involved in the control of neoplastic cells by NK cells. One is the direct release of cytolytic granules containing perforin, granzymes, and granulysin via the granule exocytosis pathway [1], [2]. Another mechanism is mediated by death-inducing ligands such as Fas ligand (FasL) and TRAIL [2], [6], [8].

TRAIL, an Apo2 ligand, is a type II transmembrane protein belonging to the TNF family. There are 5 TRAIL receptors: two can induce apoptotic signals and the others act as decoy receptors [6], [9], [10]. The ligation of TRAIL on NK cells with its two apoptotic receptors, TRAIL receptor 1 (death receptor 4) and TRAIL receptor 2 (death receptor 5), on target cells is an important mechanism of target cell lysis via the extrinsic pathway of apoptosis (as opposed to the mitochondrial pathway of apoptosis) [6], [7], [9].

Heat shock proteins (HSPs) are overproduced in many stressful conditions, including fasting. They are also involved in immune cell activation [11]–[15]. In particular, extracellular HSP70 is involved in immune stimulation [11], [14], [16], [17]. HSP70 is expressed on the surface of some tumor cells and acts as a recognition structure for NK cells, promoting NK cell cytotoxicity [18]–[20]. Furthermore, in some stressful situations, HSP70 is actively released in the extracellular space as a soluble protein or bound to exosomes to activate antigen-presenting cells [21] or NK cells [18], [22]. It has also been shown that recombinant HSP70 can stimulate the proliferation and antitumor function of NK cells [19].

Based on these studies, we hypothesized that acute starvation may lead to the enhancement of NK cell activity against neoplastic cells by inducing the expression of HSP70. In this study, we show that both the proportion of TRAIL^+^ NK cells and the expression of HSP70 were significantly elevated in the liver of fasted mice. Moreover, treatment of liver NK cells with recombinant HSP70 upregulated both TRAIL and CD69 expression, and neutralization of HSP70 in fasted mice by intraperitoneal injection of an anti-HSP70 monoclonal antibody downregulated TRAIL expression. Thus, our findings indicate that acute fasting enhances TRAIL-mediated liver NK cell activity against neoplastic cells through upregulating HSP70.

## Materials and Methods

### Ethics statements

This study was performed in strict accordance with the Guide for the Care and Use of Laboratory Animals and the local committee for animal experiments. The experimental protocol was approved by the Ethics Review Committee for Animal Experimentation of the Graduate School of Biomedical Sciences, Hiroshima University (Permit Number: A13-112). Surgery was performed under diethyl ether anesthesia, and all efforts were made to minimize the suffering of the mice.

### Mice and fasting protocol

C57BL/6J (B6) female mice aged 8–10 weeks were purchased from CLEA Japan, Inc. (Osaka, Japan). B6-based Rag-2^−/−^ γ chain^−/−^ mice aged 8–12 weeks were purchased from Taconic Farms (Hudson, NY, USA). The mice were housed in the animal facility of Hiroshima University, Japan, in a pathogen-free, microisolated environment. Prior to the start of the fasting experiments, mice were allowed *ad libitum* access to food. During the fasting experiments, the mice in the control group were allowed *ad libitum* access to food and fasted mice were deprived of food for 1 or 3 days. All mice were allowed free access to water. Mouse body weight was checked every day until the day of sacrifice. Liver weight was determined on the day of sacrifice.

### Lymphocyte isolation

After mice were anesthetized by diethyl ether, peripheral blood from the orbital sinus was collected into heparinized tubes. The peripheral blood cells (PBCs) were collected by centrifugation and red blood cells were removed using ammonium chloride potassium (ACK) lysing buffer. Liver lymphocytes were prepared according to a previously described method [23]. In brief, after injection of 1 mL phosphate-buffered saline (PBS) supplemented with 10% heparin via the portal vein, the liver was dissected out and perfused with 50 mL PBS supplemented with 0.1% ethylenediamine tetraacetic acid. Blood cells were harvested from the liver perfusate by centrifugation and erythrocytes were then removed using the ACK lysing buffer. Splenic lymphocytes were prepared as a single cell suspension by gently crushing the spleens in PBS and the erythrocytes were removed by treatment with the ACK buffer. The bone marrow cells were harvested by flushing the femurs and tibias with PBS, lymphocytes were then harvested after centrifuging and lysing red blood cells with ACK buffer. All lymphocytes were stored in RPMI medium for culture, ^51^Cr-release assay or in fluorescence-activated cell sorting (FACS) buffer to determine their phenotype by flow cytometry.

### Flow cytometric analysis

The lymphocytes were first incubated with an anti-CD16/32 (2.4G2) antibody to block nonspecific Fc-γ receptor binding and then stained with the following monoclonal antibodies (mAbs): fluorescein isothiocyanate (FITC) or BD Horizon BV421-conjugated anti-mouse NK1.1 (PK136), allophycocyanin (APC) or APC-Cy7-conjugated anti-mouse TCR-β chain (H57-597), FITC-conjugated anti-mouse CD49b (DX5) or rat IgM, k isotype control (R4-22), Alexa Fluor 647-conjugated anti-mouse CD49a (Ha31/8) or IgG2, λ1 isotype control (Ha4/8), phycoerythrin (PE)-conjugated anti-mouse CD253 (TRAIL; N2B2), CD69 (H1.2F3), CD122 (TM-Beta1), CD25 (3C7), CD314 (NKG2D; CX5), CD335 (NKp46; 29A1.4), CD178 (Fas Ligand; MFL3), or PE-conjugated mouse immunoglobulin G (IgG) 2a,k as the isotype-matched control antibody. Liver lymphocytes were also stained simultaneously with PE-Cy7 anti-mouse CD69 (H1.2F3) or PE-Cy7 Hamster IgG1, λ1 isotype control to analyze the relation between TRAIL and CD69 under fasting conditions. The apoptosis-related markers on Hepa1-6 cells were also analyzed using PE-conjugated anti-mouse CD95 (Fas/APO-1; Jo2), anti-mouse CD262 (DR5; MD5-1), anti-mouse decoy TRAIL-receptor 1 (mDcR1-3) and 2 (mDcR2-1), or PE-conjugated IgG2, λ1 isotype control antibody. All antibodies were purchased from BD Biosciences, except for CD253 (TRAIL) and CD262 (DR5) (eBioscience) and anti-mouse decoy TRAIL-receptor 1 and 2 antibodies (BioLegend). Dead cells were excluded by light scatter and propidium iodide or 7-AAD staining. Depending on the number of dyes to be detected, flow cytometric analyses were performed using the FACSCalibur (BD Biosciences), BD FACSCanto II flow cytometer (BD Biosciences), or the BD LSRFortessa X-20 (BD Biosciences). Data were analyzed using FlowJo 7.6.5 software (TreeStar, San Carlos, CA, USA).

### Isolation of NK cells and adoptive transfer assay

Liver leukocytes were obtained from wild type B6 mice. Liver NK cells were then negatively separated by using a mouse NK cell isolation kit II (Miltenyi Biotec, Auburn, CA, USA). TRAIL^−^ NK cells were further sorted magnetically using biotin-conjugated anti-mouse CD253 (TRAIL; N2B2; eBioscience) and streptavidin microbeads (Miltenyi Biotec) in the negative fraction. The purity of isolated TRAIL^−^ NK cells was assessed by flow cytometry. The liver TRAIL^−^ NK cells were intravenously injected into Rag-2^−/−^ γ chain^−/−^ mice (0.5×10^6^ cells/mouse). The transferred mice were then divided into two groups. The fasted mice received only water and fed mice received both food and water for 3 days. The lymphocytes from the liver, spleen, and bone marrow of transferred or non-transferred (control) mice were harvested after the fasting period, and NK cell phenotyping was performed.

### Cytotoxicity assay

Mouse lymphoma cells (YAC-1) and mouse hepatoma cells (Hepa1-6), both purchased from the RIKEN Cell Bank (Tsukuba, Japan), were used as the target cells. The effector cells were fresh liver lymphocytes obtained from fed (control) mice and mice that had been fasted for 3 days. The YAC-1 and Hepa1-6 cells were labeled with Na_2_[^51^Cr]O_4_ and then incubated with the effector cells in round-bottomed 96-well plates for 4 hours. The culture medium was RPMI 1640 (Gibco BRL, Grand Island, NY, USA) supplemented with 10% heat-inactivated fetal bovine serum (Sanko Chemical Co. Ltd., Tokyo, Japan), 100 IU/mL penicillin, 100 µg/mL streptomycin (Gibco BRL), 1 mM sodium pyruvate, and 1 mM nonessential amino acids (NEAA; Gibco, Grand Island, NY, USA). For the control, target cells were incubated either in culture medium to determine spontaneous release or in a mixture of 2% Nonidet P-40 to define the maximum ^51^Cr release. For the blocking assay, the effector cells were pre-incubated for 1 hour at 37°C with 50 nM concanamycin A (CMA; Sigma-Aldrich, Saint Louis, MO, USA), and/or 10 µg/mL anti-mouse CD253 (TRAIL; N2B2; eBioscience), and/or 10 µg/mL anti-mouse CD178 (FasL; MFL3; BD Biosciences), or the isotype-matched controls. Cell-free supernatants were carefully harvested and the radioactivity from the ^51^Cr that had been released into the supernatants was measured using a gamma counter (Aloka ARC-380). The cytotoxicity percentage, as indicated by ^51^Cr release, was calculated using the following equation: percent cytotoxicity =  [(cpm of experimental release – cpm of spontaneous release)]/[(cpm of maximum release – cpm of spontaneous release)] × 100.

### Western blotting

Western blotting was performed to detect HSP70, HSP27, and β-actin expression in liver tissues. For each sample, 5 mg of fresh or frozen liver tissue from either fed mice or mice that had been fasted for 3 days was homogenized in 1 mL NP-40 lysis buffer (containing 1 µL leupeptin, 1 µL aprotinin, and 10 µL 100 mM phenylmethylsulfonyl fluoride). The lysates were centrifuged at 15,000 *g* for 15 minutes at 4°C. The supernatant was then harvested, and its protein concentration was determined using a spectrophotometer (NanoDrop 2000c). The sample was then mixed with 3× SDS solution (containing 960 µL 3× Laemmli buffer and 60 µL 2-mercaptoethanol per milliliter) and boiled at 100°C for 10 minutes. For each sample, 5 mg of fresh or frozen liver tissue from either fed mice or mice that had been fasted for 3 days was homogenized in 1 mL NP-40 lysis buffer (containing 1 µL leupeptin, 1 µL aprotinin, and 10 µL 100 mM phenylmethylsulfonyl fluoride). The lysates were centrifuged at 15,000 *g* for 15 minutes at 4°C. The supernatant was then harvested, and its protein concentration was determined using a spectrophotometer (NanoDrop 2000c). The sample was then mixed with 3× SDS solution (containing 960 µL 3× Laemmli buffer and 60 µL 2-mercaptoethanol per milliliter) and boiled at 100°C for 10 minutes. For each sample, 10 µg protein was resolved by electrophoresis on 10% polyacrylamide gels with 0.1% SDS and transferred to nitrocellulose transfer membranes (Schleicher & Schuell, Keene, NH, USA), which were then incubated with an anti-HSP70 mAb (C92F3A-5; SMC-100A, StressMarq Biosciences Inc., Victoria, BC, Canada), an anti-HSP27 mAb (G3.1; ADI-SPA-800, Enzo Life Sciences), or an anti-β-actin mAb (6D1, MBL). Blots were then incubated with a peroxidase-labeled goat anti-mouse immunoglobulin antibody (NA 931; Amersham International, Buckinghamshire, UK) and developed using X-ray film and an enhanced chemiluminescence detection reagent (Amersham Pharmacia Biotech). The band density on the X-ray film was quantified using ImageJ software (NIH, Bethesda, MD, USA).

### Treatment of liver lymphocytes with recombinant HSP70

Liver lymphocytes (2 million/well) isolated from fed B6 mice were cultured with recombinant mouse HSP70-A1 (ADI-SPP-502, Enzo Life Sciences) at different concentrations (0 µg/mL as the control or 0.5, 5, and 50 µg/mL) with or without 20 ng/mL recombinant mouse interleukin (IL)-2 (eBioscience) in RPMI 1640 supplemented with 10% fetal bovine serum, 100 IU/mL penicillin, 100 µg/mL streptomycin (Gibco BRL, Carlsbad, CA, USA), 1 mM sodium pyruvate, and 1 mM NEAA (Gibco BRL). After 2, 3, or 5 days of culture, the lymphocytes were harvested and the NK cell phenotype was analyzed by flow cytometry.

### HSP70 inhibition by anti-HSP70 antibody *in vivo*

Eight-week-old B6 female mice were intraperitoneally injected with 200 µL PBS containing 100 µg of either mouse anti-HSP70 mAb (clone C92F3A-5; without sodium azide; StressMarq Biosciences Inc.) or a mouse IgG isotype-matched control antibody (Jackson ImmunoResearch Laboratories Inc.) just before fasting (6 mice per group). Mouse body weight was measured every day. After fasting for 3 days, the mice were sacrificed, and their liver lymphocytes were harvested to determine TRAIL and CD69 expression on NK cells by flow cytometry.

### Statistical analysis

Data are presented as mean plus standard deviation or standard error of the mean. The statistical differences between 2 groups were analyzed using an independent samples T test (2-tailed) in SPSS Statistics version 16.0 (IBM, Rockford, IL, USA); *p-*values of 0.05 or less were considered to indicate significance.

## Results

### The proportion of TRAIL^+^ and CD69^+^ NK cells increased in mouse livers in response to starvation

The phenotypic characteristics of NK cells in mice that had been fasted for 1–3 days were examined by flow cytometry. Liver lymphocytes from both fed and fasted mice were harvested and stained with various antibodies to identify the membrane markers TRAIL, CD69, CD122 (IL-2 receptor β chain), and CD25 (IL-2 receptor α chain).

**Figure 1:**
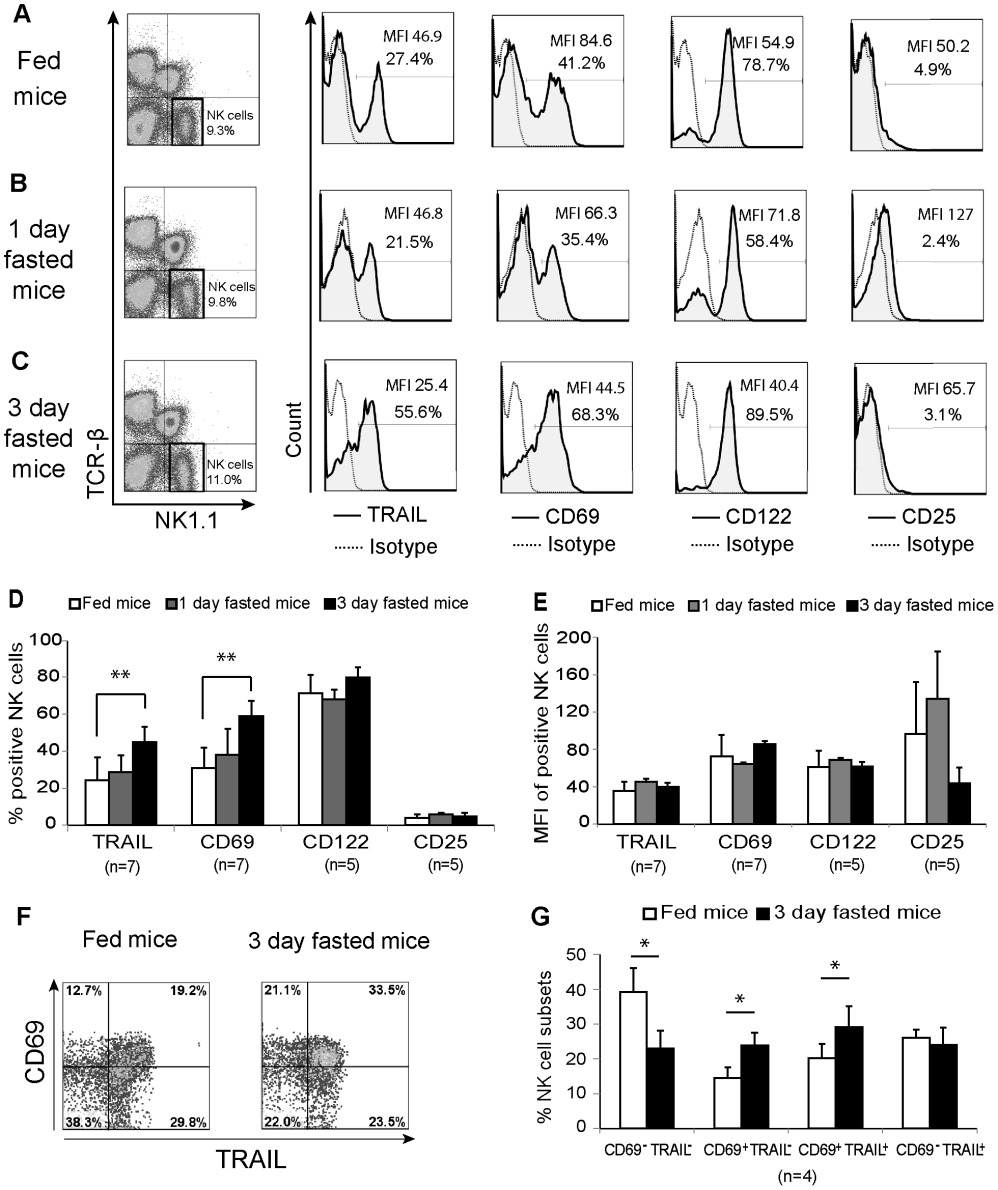
Phenotype of liver natural killer cells under starvation. Isolated liver lymphocytes from 3 mouse groups were stained with monoclonal antibodies against the cell surface markers TRAIL, CD69, CD122, and CD25 prior to analysis by flow cytometry. Representative natural killer (NK) cell phenotype analyses from (A) fed, (B) 1-day-fasted and (C) 3-day-fasted mice are presented in dot plots and histograms. TCRβ^−^ NK1.1^+^ cells were gated as NK cells. The dotted lines represent the negative control. The distribution of TRAIL, CD69, CD122, and CD25 expression in NK cells is indicated by the solid lines (shaded areas) and the percentage and mean fluorescence intensity (MFI) of positive cells are provided. (D) The percentages or (E) MFI of liver NK cells that are positive for TRAIL, CD69, CD122, and CD25 are shown in bar graphs as mean plus standard deviation. (F) Dot plots of representative data and (G) bar graph present the mean plus standard deviation of proportion of NK cell subsets regarding to TRAIL and CD69 expression in fed and fasted mice; *p <0.05, **p <0.01 as analyzed by the independent samples T test.

Electronically gated TCRβ^−^ NK1.1^+^ NK cells and NK cell markers from a representative fed, 1-day-fasted, or 3-day-fasted mouse are shown in Figure 1 A–C. Notably, compared to fed mice, 3-day-fasted mice showed significantly higher proportions of TRAIL and CD69 in liver NK cells (Figure 1D). Mean fluorescence intensity (MFI) of TRAIL or CD69 positive NK cells showed no significant differences among the groups (Figure 1E). Next, the distribution analysis of CD69 and TRAIL expression revealed that the proportion of CD69^+^TRAIL^+^ double positive NK cells significantly increased in fasted mice, while CD69^−^ TRAIL^−^ NK cells significantly decreased. The proportion of CD69^+^ TRAIL^−^ cells also increased (Figure 1F, G). There was no difference in CD122 and CD25 expression in liver NK cells among the groups (Figure 1D, E) as well as in splenic NK cells (data not shown). The proportion of NK cells in the liver mononuclear cell fractions from 3-day-fasted mice did not differ from that from fed mice (Figure S1A).

**Figure 2:**
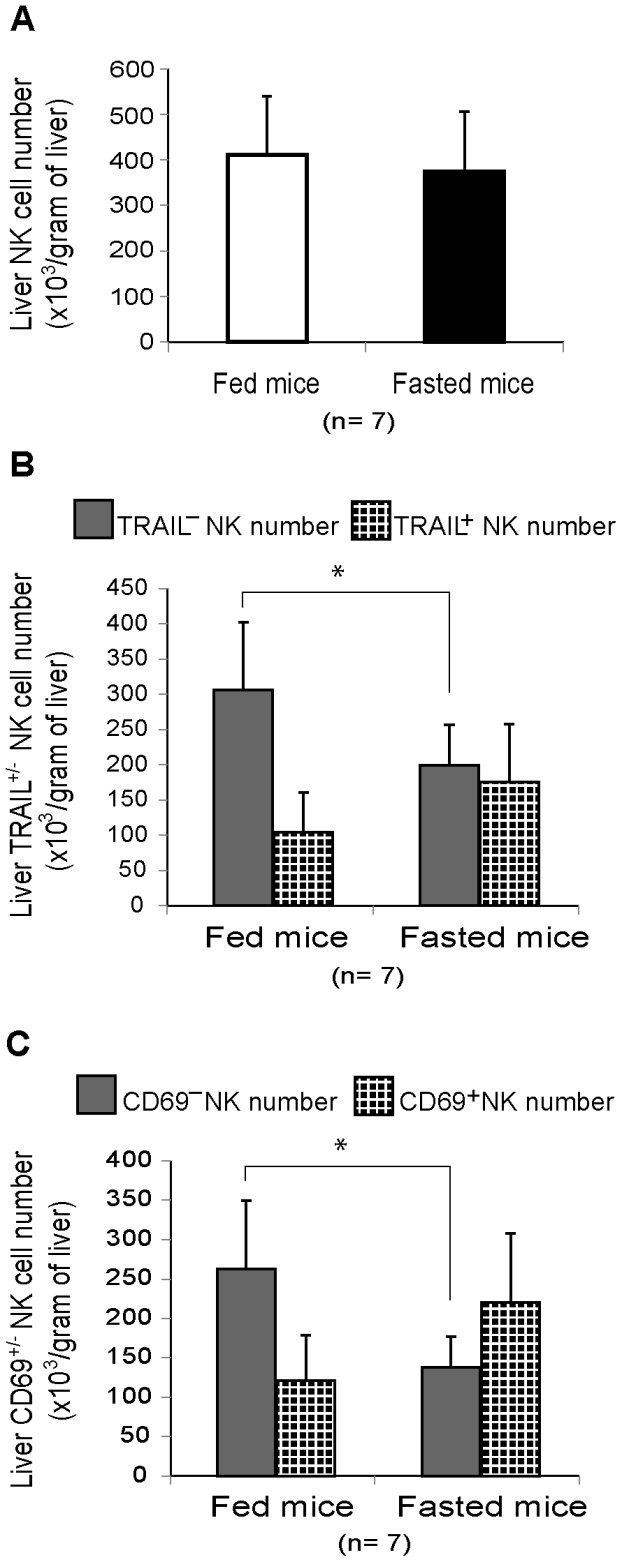
Distribution of TRAIL and CD69 expression in liver natural killer cells in response to starvation. Liver lymphocytes from fed mice and 3-day-fasted mice were stained with monoclonal antibodies and counted using flow cytometry. Numbers of (A) TCRβ^−^ NK1.1^+^ natural killer (NK) cells, (B) TRAIL^+/−^ NK cells, and (C) CD69^+/−^ NK cells per gram of liver tissue are presented in bar graphs as mean plus standard deviation (n = 7); *p <0.05 as analyzed by the independent samples T test.

The influence of fasting on the absolute number of NK cells was also examined. The total number of liver resident NK cells in a unit weight of liver tissue did not differ between fasted and fed mice, indicating that, under fasting conditions, the number of TRAIL^−^ NK cells decreased, while that of TRAIL^+^ NK cells increased (Figure 2A, B).

**Figure 3:**
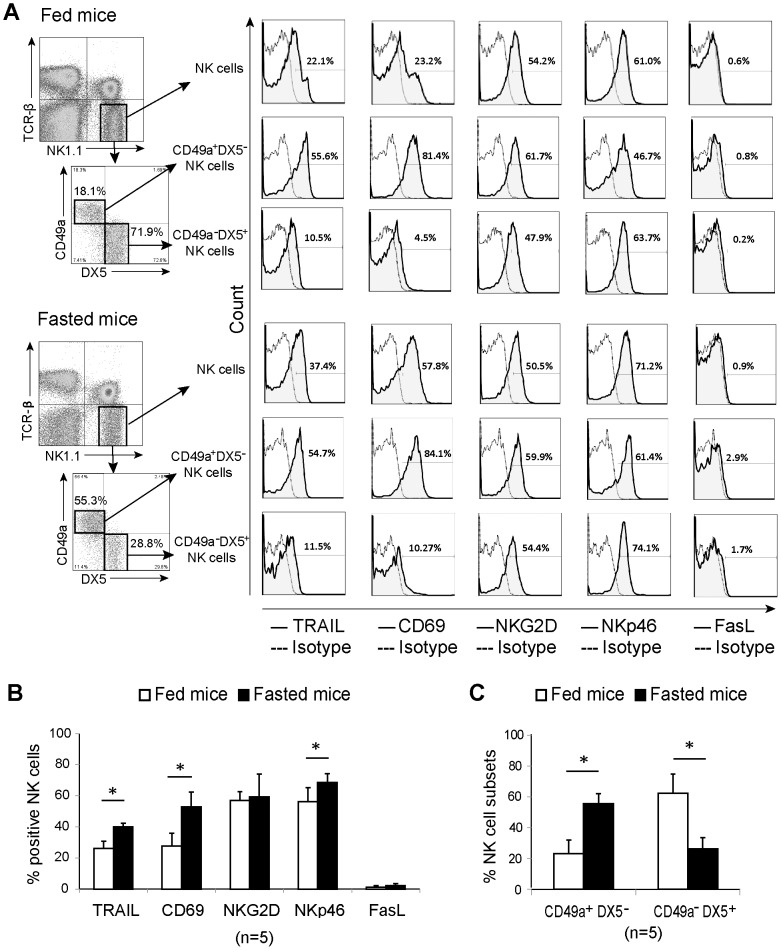
Analysis of functional markers in liver natural killer cells and their CD49a^+^ DX5^−^ and CD49a^−^ DX5^+^ subgroups. Liver lymphocytes from fed and 3-day-fasted mice were simultaneously stained with monoclonal antibodies against DX5, CD49a, TRAIL, CD69, NKG2D, NKp46, and FasL. (A) Representative dot plots of gated TCRβ^−^ NK1.1^+^ natural killer (NK) cells and its two subsets, CD49a^+^ DX5^−^ and CD49a^−^ DX5^+^ NK cells, in fed and fasted mice are presented. Histograms show the expression of TRAIL, CD69, NKG2D, NKp46, and FasL (solid lines) on whole NK cells and their subsets with the percentage of NK cells that are positive for those markers, dotted lines present negative control. (B) Bar graph shows the mean percentage plus standard deviation of NK cells that are positive for TRAIL, CD69, NKG2D, NKp46, or FasL. (C) The proportion of NK cell subsets, CD49a^+^ DX5^−^ and CD49a^−^ DX5^+^ NK cells, in fed and fasted mice are shown as mean ratio plus standard deviation; *p <0.05 as analyzed by the independent samples T test.

Analysis of other functional markers of NK cells indicated that whole liver NK cells from 3-day fasted mice highly expressed not only TRAIL and CD69 but also NKp46 when compared with fed mice. There was no significant difference in NKG2D or FasL expression (Figure 3A, B). Additionally, changes in CD49a and DX5 phenotype characteristics in NK cells were examined based on a report that recently demonstrated that liver-resident CD3^−^ NK1.1^+^ NK subsets are characterized according to the differential expression of CD49a and DX5 [24]. While proportions of CD49a^−^ DX5^+^ NK cells significantly decreased in fasted mice, the proportion of CD49a^+^ DX5^−^ NK cells, which highly expressed TRAIL and CD69, significantly increased (Figure 3A, C). Taken together, our results indicate that, under 3-day fasting, TRAIL and CD69 are highly expressed in mouse liver-resident CD49a^+^ DX5^−^ NK cells.

The phenotypic characteristics of NK cells in the spleen, bone marrow, and peripheral blood were also examined under starvation (Figure S1A-G). The proportion of NK cells did not differ in the spleen as well as in the liver, while it increased in the bone marrow and decreased in peripheral blood under fasting conditions (Figure S1A). Splenic NK cells from fasted mice showed a trend similar to that of liver NK cells in terms of CD69 expression, but NK cells in other organs did not show a similar trend. Unlike the findings for the liver, the spleens from both fed and fasted mice presented a very low CD49a^+^ DX5^−^ NK cell fraction (data not shown).

### TRAIL upregulation on liver NK cells in adoptive transferred fasted mice

**Figure 4:**
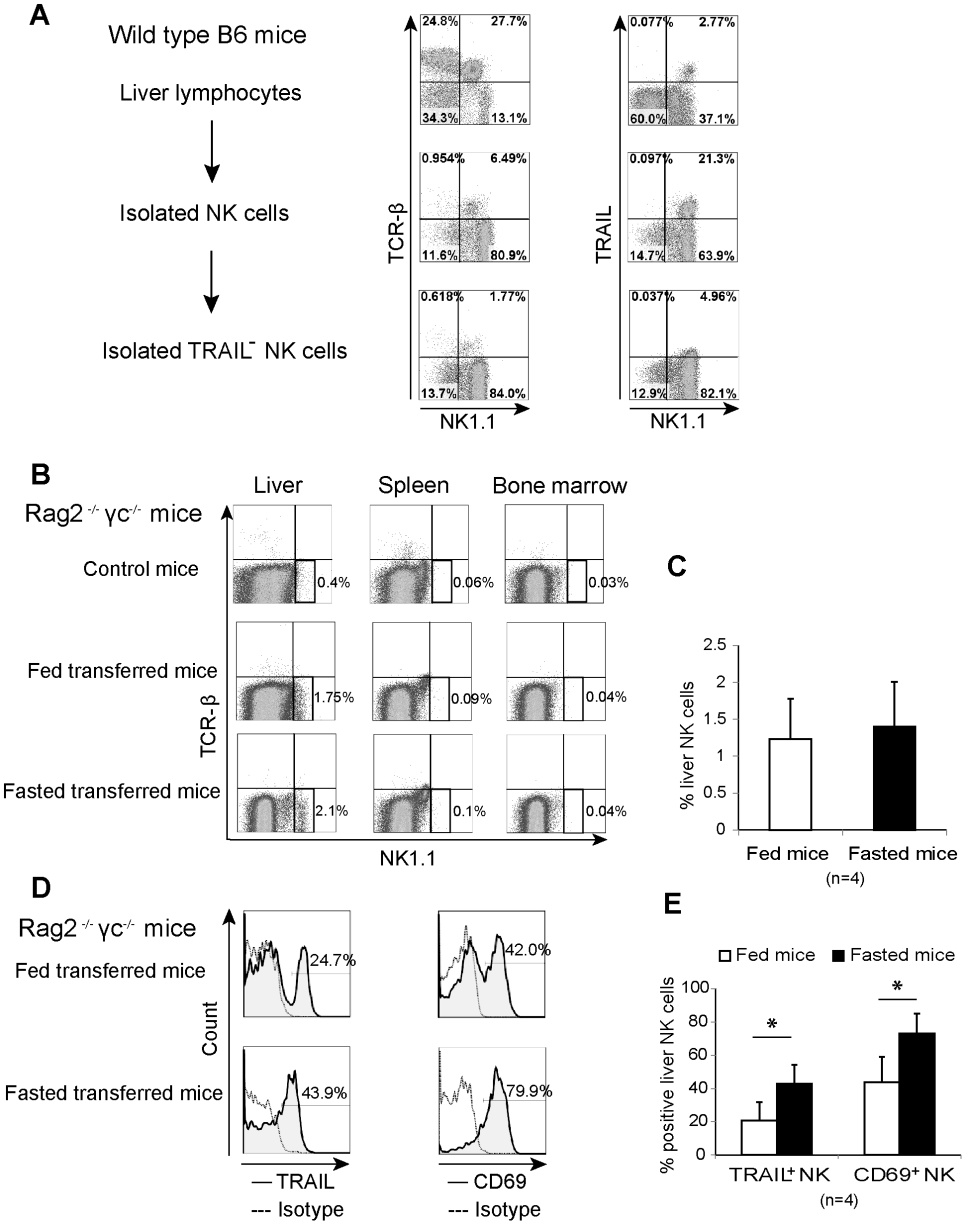
Adoptive transfer assay for TRAIL and CD69 expression on liver natural killer cells in response to starvation. (A) Isolated TRAIL^−^ natural killer (NK) cells were separated from liver lymphocytes of wild type B6 mice. Proportion of lymphocytes expressing TCRβ, NK1.1, and TRAIL in whole liver lymphocytes, isolated NK cells, and isolated TRAIL^−^ NK cells are presented in dot plots. The isolated TRAIL^−^ NK cells were adoptively transferred into Rag-2^−/−^ γ chain^−/−^ mice (0.5×10^6^ cells/mouse), which were then fed or fasted for 3 days before determining their NK phenotype. (B) Dot plots show the gated TCRβ^−^ NK1.1^+^ NK cells and their percentage in the liver, spleen, and bone marrow of non-transferred (control), fed-transferred, and fasted-transferred mice. (C) Bar graph presents the mean percentage plus standard deviation of NK cells in the liver of fed and fasted-transferred mice. (D) Expression of TRAIL and CD69 (solid lines) on the liver NK cells of representative fed- and fasted-transferred mice with their percentages of positive cells are presented in histograms; dotted lines showed the negative control. (E) The proportion of liver TRAIL^+^ and CD69^+^ NK cells in fed and fasted-transferred mice are shown in bar graph as mean plus standard deviation; *p <0.05 as analyzed by the independent samples T test.

We next examined the mechanism of TRAIL upregulation in fasted mice. To clarify whether TRAIL^−^ NK cells convert into TRAIL^+^ NK cells in fasting mice, we transferred TRAIL^−^ NK cells that were isolated from liver lymphocytes obtained from wild type B6 mice into Rag-2^−/−^ γ chain^−/−^ B6 mice. The NK cell purity and TRAIL expression rate on the isolated NK cells are shown in Figure 4A. It is noteworthy that these mice present macrophages, but not NK cells or other lymphocytes. The absence of NK cells in Rag-2^−/−^ γ chain^−/−^ mice was analyzed in Figure 4B (control mice). Three days after injection, the injected NK cells homed to the liver, but not to the spleen or the bone marrow (Figure 4B, C). Furthermore, fasted transferred mice showed significantly high expression of TRAIL and CD69 in liver NK cells in comparison with fed transferred mice (Figure 4D, E). These results indicate that TRAIL upregulation is induced in liver-resident NK cells by converting TRAIL^−^ cells into TRAIL^+^ cells.

### Cytotoxicity of liver lymphocytes against TRAIL-sensitive cancer cells increased in fasted mice

**Figure 5:**
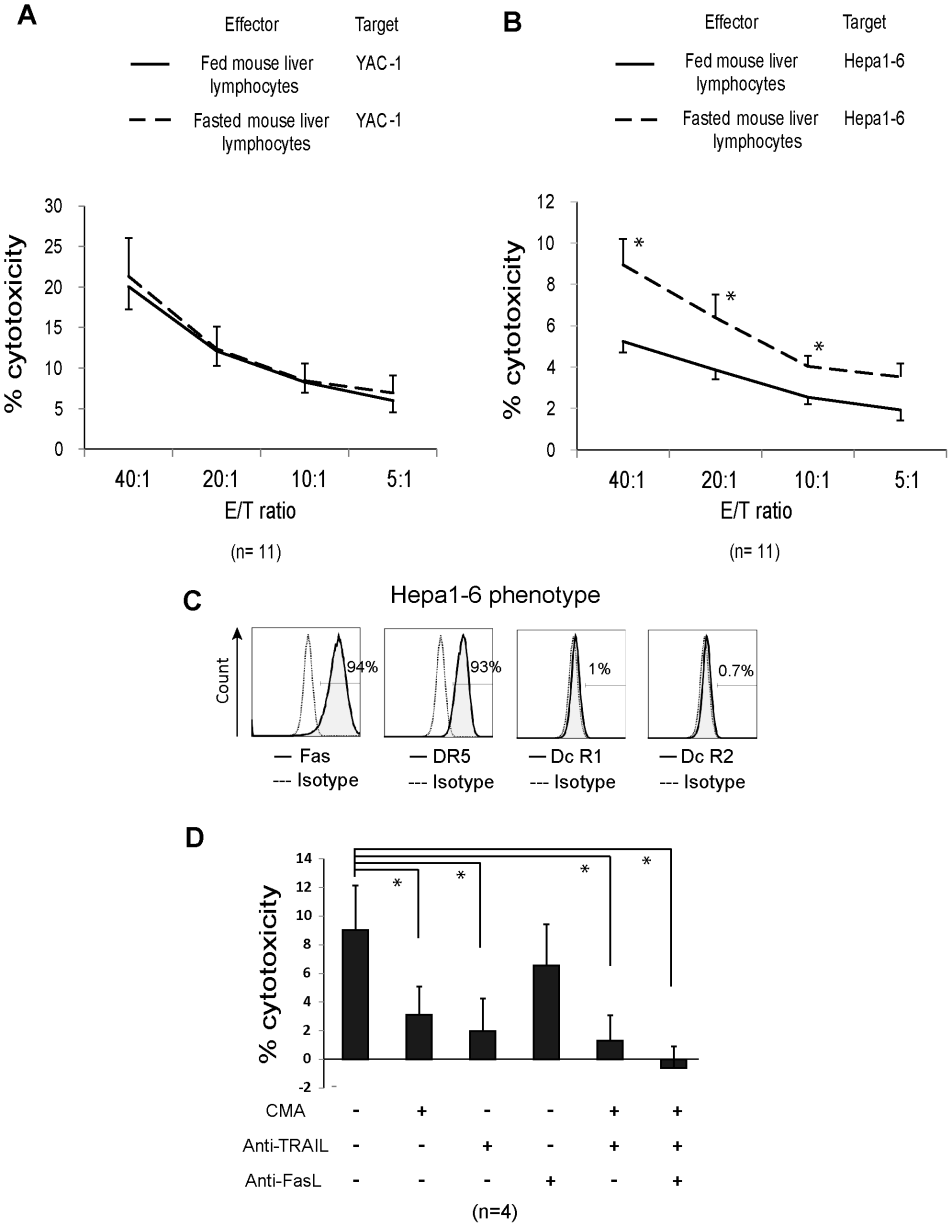
Assay analyzing cytotoxic effects of liver lymphocytes obtained from fasted mice on tumor cells. (A) The cytotoxic activity of freshly isolated liver lymphocytes from fed mice (solid lines) and 3-day-fasted mice (dashed lines) against TRAIL-resistant YAC-1 and (B) TRAIL-sensitive Hepa1-6 cells was analyzed using the ^51^Cr-release assay. The effector to target (E/T) ratios were 40∶1, 20∶1, 10∶1, and 5∶1. The cytotoxicity percentage was calculated as the percentage of specific ^51^Cr release, as described in the materials and methods section. Data are presented as mean ± standard error of the mean from triplicate samples of 11 repeated assays, each including 1 fed and 1 fasted mouse. (C) Histograms show the phenotype of Hepa1-6 cells that was analyzed using antibodies against mouse Fas, death receptor 5 (DR5), and decoy TRAIL receptor 1 and 2 (DcR1 and DcR2) in solid lines. Negative controls, which were stained with isotype-math antibodies, are indicated using dotted lines. The proportion of Hepa1-6 cells positive for those markers is provided. (D) Liver lymphocytes that were obtained from 3-day-fasted mice were incubated with CMA, anti-TRAIL mAb, anti-FasL mAb, or their combination before incubation with ^51^Cr-labeled-Hepa1-6 for 4 hours, at a lymphocyte: Hepa1-6 ratio of 40∶1. Bar graph shows the mean cytotoxicity percentage plus standard deviation for each group. Statistical analysis was performed for each ratio using the independent samples T test; *p <0.05.

The cytotoxic potential of NK cells against the cell lines YAC-1 and Hepa1-6, which differ in their sensitivity to TRAIL, was determined using the ^51^Cr release assay. Liver lymphocytes from fed and 3-day-fasted mice were used as the effectors. There was no difference in the cytotoxicity of the two lymphocyte groups against TRAIL-resistant YAC-1 (Figure 5A). However, liver lymphocytes from fasted mice showed significantly higher cytotoxicity against TRAIL-sensitive Hepa1-6 than liver lymphocytes from fed mice at effector: target ratios of 40∶1, 20∶1, and 10∶1 (Figure 5B). To further investigate whether the upregulated cytotoxicity was mediated via TRAIL, we incubated liver lymphocytes from fasted mice with perforin inhibitor (CMA), anti-TRAIL mAb, anti-FasL mAb, or their combination at an effector: target ratio of 40∶1. Hepa1-6 receptor expression was also examined. Hepa1-6 cells highly expressed both Fas and death receptor 5 (TRAIL receptor 2) (Figure 5C). Lymphocytes treated with CMA, anti-TRAIL mAb, or their combination presented a significantly reduced cytotoxicity in comparison with the untreated group (Figure 5D). In contrast, the group treated with anti-FasL showed no significant difference. These results indicate that liver NK cells from fasted mice presented an increased perforin- and TRAIL-mediated antitumor activity.

### Overexpression of HSP70 was induced in livers from fasted mice

**Figure 6:**
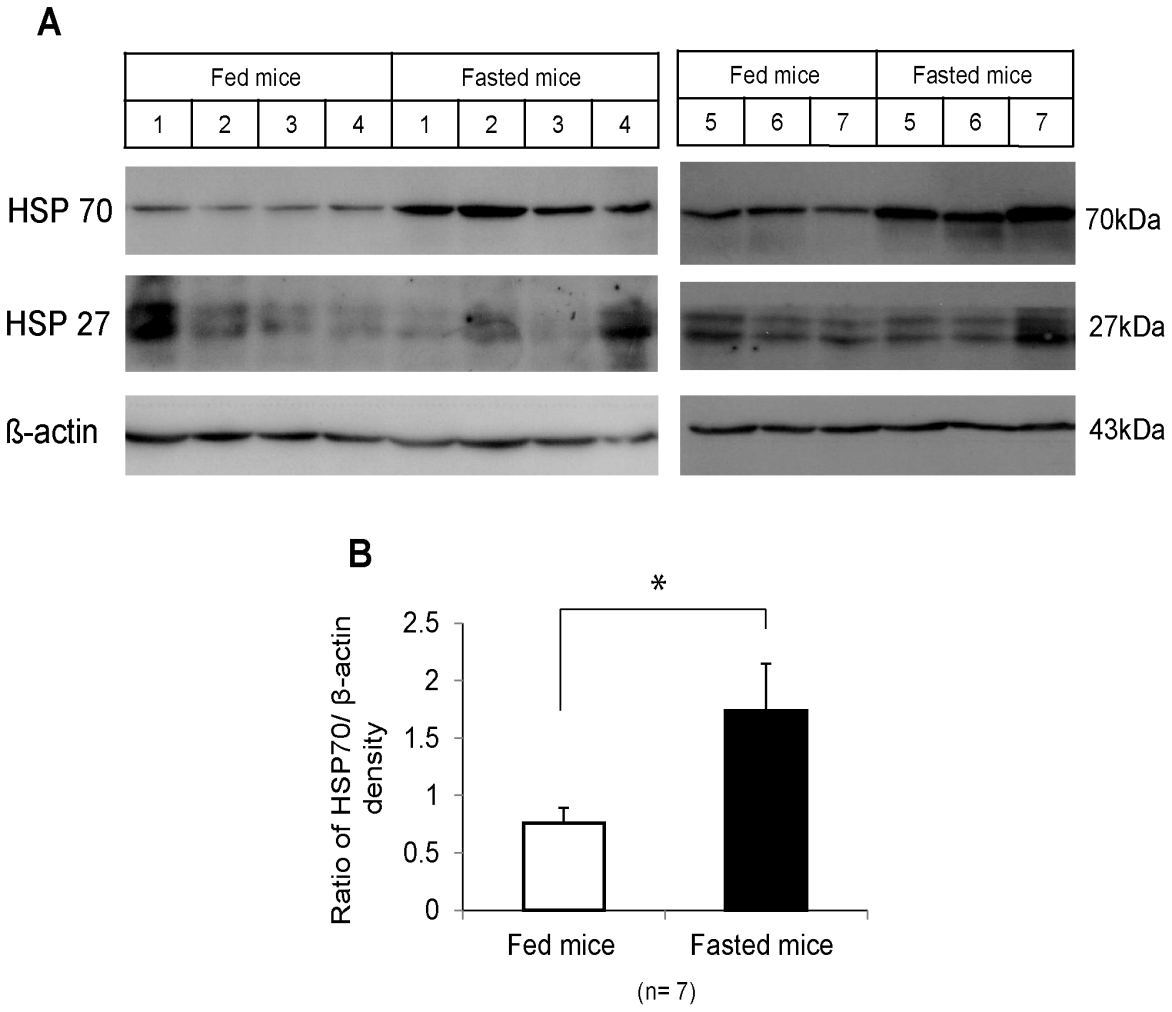
Western blot analysis of heat shock protein expression in fasted mouse livers. (A) Heat shock protein (HSP)70, HSP27, and β-actin expression in the livers from fed mice (control) and 3-day-fasted mice (7 mice in each group) was determined by western blot. (B) The bar graph shows the average HSP70/β-actin densities plus standard error of the mean; densities were analyzed using ImageJ software. Statistical analyses were performed using the independent samples T test. *p <0.05.

It has been demonstrated that HSP70 actively released in the extracellular space activates NK cells [18], [22]. Hence, HSPs induced by acute starvation may play a role in TRAIL-mediated antitumor activity. We found that HSP70 expression was significantly higher in 3-day-fasted mouse liver than in fed mouse liver (*p* <0.05), while HSP27 expression was not changed (Figure 6).

### Treatment with recombinant HSP70 induced the proliferation and activation of liver NK cells

**Figure 7:**
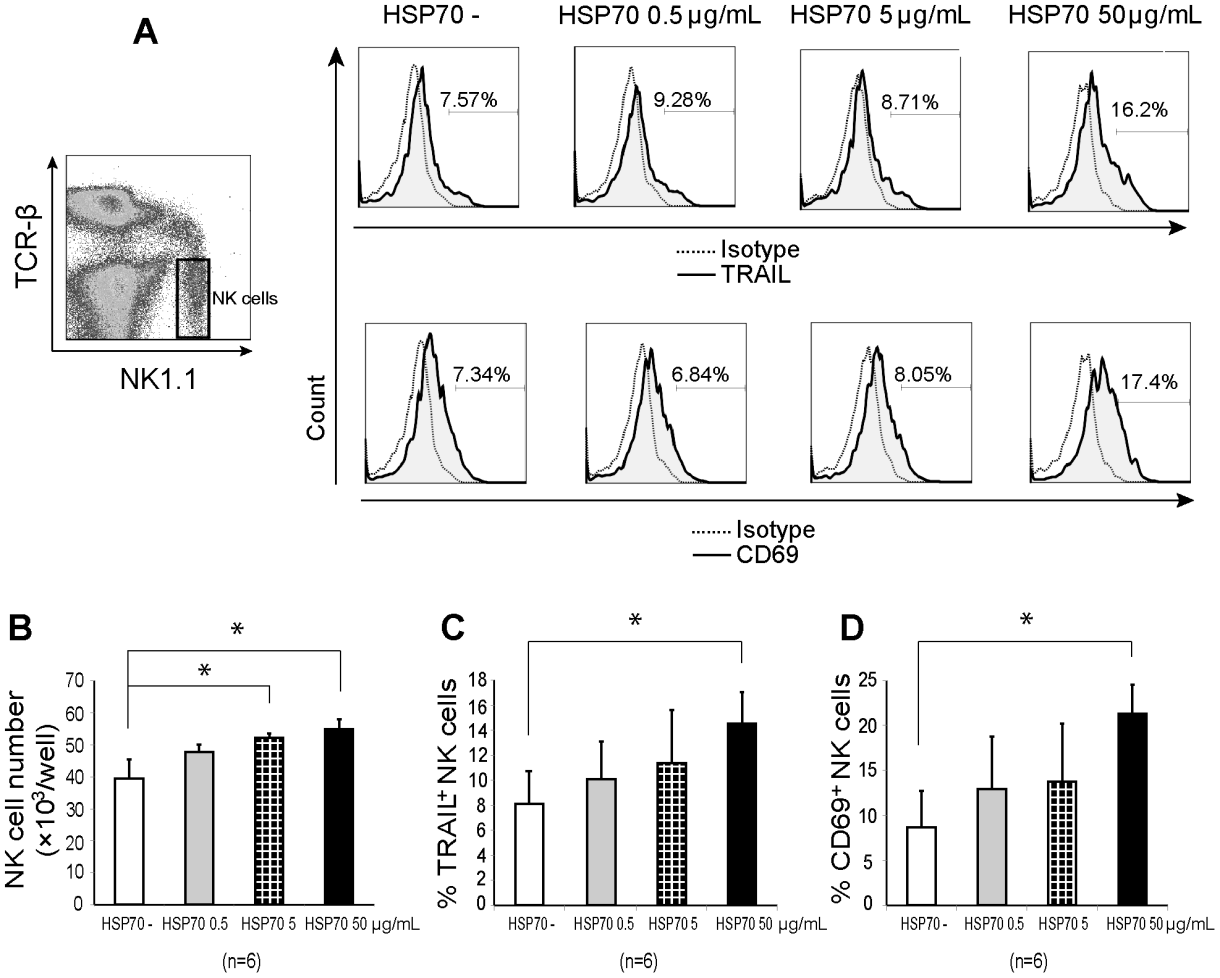
The effect of recombinant heat shock protein 70 on natural killer cell proliferation and TRAIL and CD69 expression. Isolated liver lymphocytes (2 million cells/well) were cultured with mouse recombinant heat shock protein (HSP) 70 at various concentrations: 0, 0.5, 5, or 50 µg/mL. After 3 days of culture, the lymphocytes were harvested for phenotypic determination. (A) Representative flow cytometric analysis of TRAIL and CD69 expression in TCRβ^−^ NK1.1^+^ natural killer (NK) cells is shown. The dotted lines represent the expression distribution in the negative control cells and the solid lines (shaded areas) with numbers indicate the distribution of TRAIL^+^ and CD69^+^ NK cells. (B) TCRβ^−^ NK1.1^+^ NK cell number per well, (C) TRAIL^+^ and (D) CD69^+^ NK cell percentages are shown in bar graphs as mean plus standard deviation (n = 6). Data were statistically analyzed using the independent samples T test; *p <0.05.

The contribution of HSP70 to NK cell activation was assessed *in vitro* by examining the phenotypic characteristics of mouse liver NK cells after culturing liver lymphocytes with IL-2 and different concentrations of recombinant HSP70 (0 µg/mL as the control, 0.5, 5, or 50 µg/mL) for 3 days. Treatment with ≥5 µg/mL HSP70 induced NK cell proliferation (*p* <0.05; Figure 7B), whereas treatment with 50 µg/mL HSP70 led to an upregulation of TRAIL and CD69 expression in liver NK cells as compared to the control (Figure 7C, D).

### Anti-HSP70 neutralizing antibody reduced TRAIL expression in liver NK cells in fasted mice

**Figure 8:**
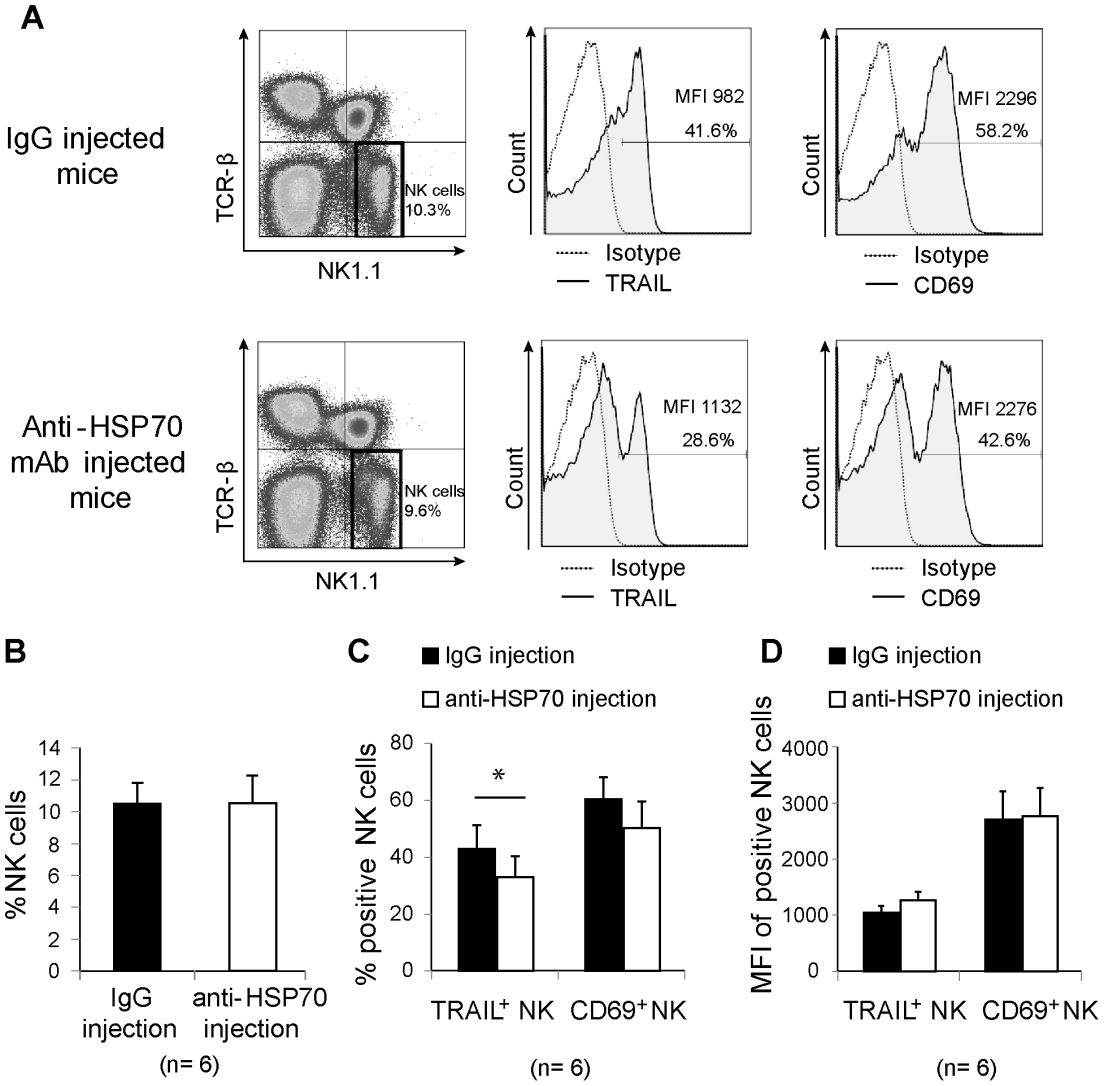
Neutralization effect of an anti-heat shock protein 70 monoclonal antibody on the natural killer cell phenotype. Mice received intraperitoneal injections of an anti-heat shock protein (HSP) 70 monoclonal antibody or isotype-matched mouse immunoglobulin G (6 mice per group) just before fasting. After a 3-day fast, liver lymphocytes were harvested for phenotyping by flow cytometry. (A) The distribution of TRAIL and CD69 expression on electronically gated TCRβ^−^ NK1.1^+^ natural killer (NK) cells is indicated with solid lines (shaded areas). The percentage and mean fluorescence intensity (MFI) of TRAIL^+^ or CD69^+^ NK cells are provided. The dotted lines represent the distribution in the negative control. (B) TCRβ^−^ NK1.1^+^, (C) TRAIL^+^ and CD69^+^ NK cell proportions, and (D) MFI from NK cells positive for those markers are shown in bar graphs as mean plus standard deviation (n = 6). The difference among the groups was analyzed using the independent samples T test; *p <0.05.

To further clarify the relationship between HSP70 and TRAIL-mediated NK cell function, an *in vivo* HSP70 neutralization assay was performed. Either an anti-HSP70 mAb or a mouse IgG isotype-matched control antibody was intraperitoneally injected (100 µg per mouse) into mice on day 0 before fasting. After the mice had been fasted for 3 days, their liver lymphocytes were harvested for NK cell phenotypic determination. The two groups of mice did not differ in terms of their body weight or liver lymphocyte yield (data not shown). TRAIL and CD69 expression in the TCRβ^−^ NK1.1^+^ NK cells was then assessed by flow cytometry (Figure 8A). Although there was no difference in NK cell frequency between the two groups (Figure 8B), TRAIL expression in liver NK cells from mice injected with the anti-HSP70 mAb was significantly lower than that in cells from the control group (Figure 8C). CD69 expression was also downregulated in cells from mice injected with the anti-HSP70 mAb, but no significance was observed (*p* = 0.07; Figure 8C). MFI of TRAIL or CD69 positive NK cells did not significantly differ between two groups of mice (Figure 8D).

## Discussion

Acute starvation is well known to induce physiological changes in the body. Consistent with previous studies [4], [25], our study showed a decrease in body weight and liver weight as well as in the number of lymphocytes from various organs in fasted mice as compared to fed mice (Figure S2A–H). Interestingly, we observed that, although the liver weight decreased proportionately with body weight (i.e., the liver:body weight ratio was unchanged), the lymphocyte number notably decreased under starvation.

We previously reported that liver NK cells constitute a unique NK population characterized by high TRAIL expression and high production of perforin, granzymes, and cytokines and have the capacity to kill various kinds of cancer cells, virus-infected cells, or other transformed cells [26], [27]. The ligation of TRAIL with death receptor 4 or 5 on target cells induces NK cell activation [7]. On the other hand, CD69, which is a type II transmembrane glycoprotein, is highly induced in many activated lymphocytes, in particular in NK cells [28]. This study represents the first report showing that the proportion of liver-resident NK cells expressing TRAIL and CD69 is significantly higher in fasted mice than in fed mice (Figures 1 and 3). The adoptive transfer assay indicated that TRAIL^−^ NK cells could turn into TRAIL^+^ NK cells under fasting condition (Figure 4). Taken together with the fact that the total number of liver resident NK cells, including both TRAIL^+^ and TRAIL^−^, in a unit weight of liver tissue did not differ between fasted and fed mice (Figure 2), our results confirm that fasting leads to the activation of liver NK cells.

Liver NK cells from fasted mice have previously been demonstrated to have high antitumor activity [4]. However, the mechanism underlying this activity has been entirely unknown. Our study indicates that liver lymphocytes from fasted mice showed high cytotoxicity against TRAIL-sensitive Hepa1-6 cells and related to the TRAIL-mediated apoptotic pathway (Figure 5). Furthermore, these lymphocytes contained a higher proportion of TRAIL^+^ NK cells than those from fed mice (Figure 1D). In contrast, TRAIL expression in other kind of lymphocytes such as T cells and NKT cells was very low and did not differ between fed and fasted mice (data not shown). These observations suggest that the cytoxicity in liver NK cells from fasted mice is linked to the specific upregulation of TRAIL by acute starvation.

Our result may help understand the innate immune response in post-operative fasted and cachectic patients or patients with other conditions suffering from fasting. Besides many negative effects of starvation, such as fatigue and weight loss, fasting may still exert high level of antitumor effects via TRAIL-mediated NK cell activity. This might provide a new therapeutic approach to activate TRAIL-mediated NK cell activity in patients; further studies are needed in this regard.

Many factors contribute to the regulation of TRAIL expression in NK cells. Interferon gamma (IFN-γ) is one of the most important factor, which can both induce TRAIL expression in NK cells and mediate NK cell cytotoxic activity [8], [29], [30]. Other cytokines such as IL-2, IL-12, IL-15, IL-18, and IL-21 have been shown to be involved in the survival and antitumor activity of NK cells [31]. However, neither IFN-γ nor IL-12 is upregulated in 3-day-fasted mice [4], and neither IL-12 nor IL-18 induced TRAIL expression in liver NK cells [30].

It is well known that HSPs are strongly induced in various stressful situations to cope with stimuli. HSP60 and GRP78 were found to be induced in response to fasting [13], [32]. In this study, we found that HSP70 was significantly overexpressed in the liver of fasted mice (Figure 6). HSP70 can actively translocate into the plasma membrane following some stresses and even be released into the extracellular space to stimulate immune cells [21], [33].

Previous studies have shown that HSP70 is linked to NK cell cytotoxicity. Membrane-bound HSP70 on tumor cells has been identified as a recognition structure for NK cells that promotes NK cell cytotoxicity [18]–[20], [34], and an *in vitro* study has shown that culturing NK cells with HSP70 leads to an increase in their cytotoxicity [19], [20]. In addition, adoptive infusion of HSP70/IL-2 pre-stimulated NK cells induced shrinking of tumor masses in tumor-bearing mice and improved survival [18]–[20], [35]. Despite such striking facts, it is still not fully understood which molecules are responsible for NK cell immunostimulatory response to HSP70.

In the present study, we cultured recombinant HSP70 with liver lymphocytes and found that NK cell proliferation increased with HSP70 stimulation (Figure 7B). Furthermore, both TRAIL and CD69 expression in liver NK cells from fed mice were upregulated in response to HSP70 in a dose-dependent manner (Figure 7C, D). This result suggests that HSP70 may play a role in the stimulation of TRAIL expression in NK cells during fasting. Thus, to determine the effect of HSP70 on TRAIL expression, HSP70 inhibition using an anti-HSP70 mAb was performed *in vivo*. As expected, TRAIL^+^ NK cell proportion was significantly downregulated in the anti-HSP70 mAb–treated mice. To our knowledge, this is the first report to provide evidence that HSP70 can induce NK cell activation in fasted mice via TRAIL. Since TRAIL downregulation by HSP70 inhibition is not complete, there may be other factors that regulate TRAIL-mediated NK activity; further studies are needed in this regard.

In conclusion, our mouse-model study showed that starvation has a positive effect on innate immunity by activating liver NK cells through TRAIL upregulation. We also showed that the underlying mechanism is, at least in part, due to HSP70 overexpression in the liver. This insight into HSP70-mediated NK cell activation may lead to the development of new therapeutic approaches that use NK cells to target cancer or virus-infected cells.

## Supporting Information

Figure S1 **Additional phenotypic analysis of natural killer cells from the spleen, bone marrow, and blood under starvation.** (A) The mean proportion plus standard deviation of gated TCRβ^−^ NK1.1^+^ natural killer (NK) cells from the liver, spleen, bone marrow, and blood of fed and 3-day-fasted mice are shown in bar graphs. (B) Histograms show the representative expression of the indicated markers on NK cells (solid lines) with the percentages of positive NK cells from the spleen, (D) bone marrow, and (F) blood; dotted lines represent negative control. Bar graphs represent the mean percentage plus standard deviation of positive NK cells in (C) the spleen, (E) bone marrow, and (G) blood. Data were analyzed using the independent samples T test; *p <0.05.
(TIF)

Figure S2 **Physiological characteristics of the fasted mice.** (A) Mouse body weight was measured every day during the fasting period. (B, C) Liver weight and ratio of liver:body weight were determined on the day of sacrifice. Lymphocytes from (D, E) the liver, (F) spleen, (G) bone marrow, and (H) blood from fed and fasted mice were counted using a hemocytometer; average numbers plus standard deviation are shown. The difference between groups was analyzed using the independent samples T test; *p <0.05; **p <0.01.
(TIF)
