# Supplementary figures and images for: Fasting Enhances TRAIL-Mediated Liver Natural Killer Cell Activity via HSP70 Upregulation
Source: PLoS One. 2014 Oct 30;9(10):e110748. doi: 10.1371/journal.pone.0110748 (PMC4214715; doi:10.1371/journal.pone.0110748)

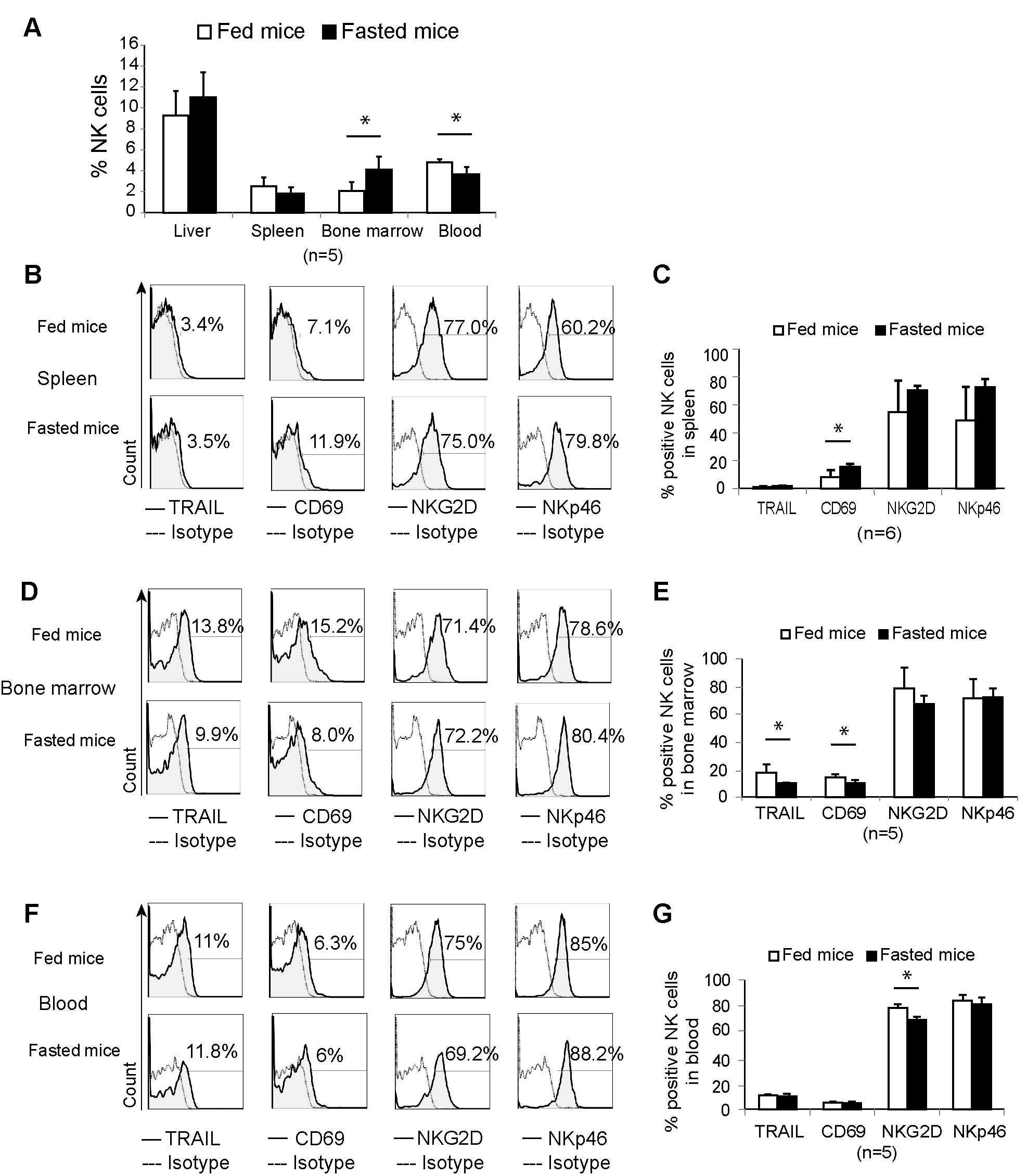

Supplement: Figure S1 — Additional phenotypic analysis of natural killer cells from the spleen, bone marrow, and blood under starvation. (A) The mean proportion plus standard deviation of gated TCRβ− NK1.1+ natural killer (NK) cells from the liver, spleen, bone marrow, and blood of fed and 3-day-fasted mice are shown in bar graphs. (B) Histograms show the representative expression of the indicated markers on NK cells (solid lines) with the percentages of positive NK cells from the spleen, (D) bone marrow, and (F) blood; dotted lines represent negative control. Bar graphs represent the mean percentage plus standard deviation of positive NK cells in (C) the spleen, (E) bone marrow, and (G) blood. Data were analyzed using the independent samples T test; *p <0.05. (TIF) [file pone.0110748.s001.tif]

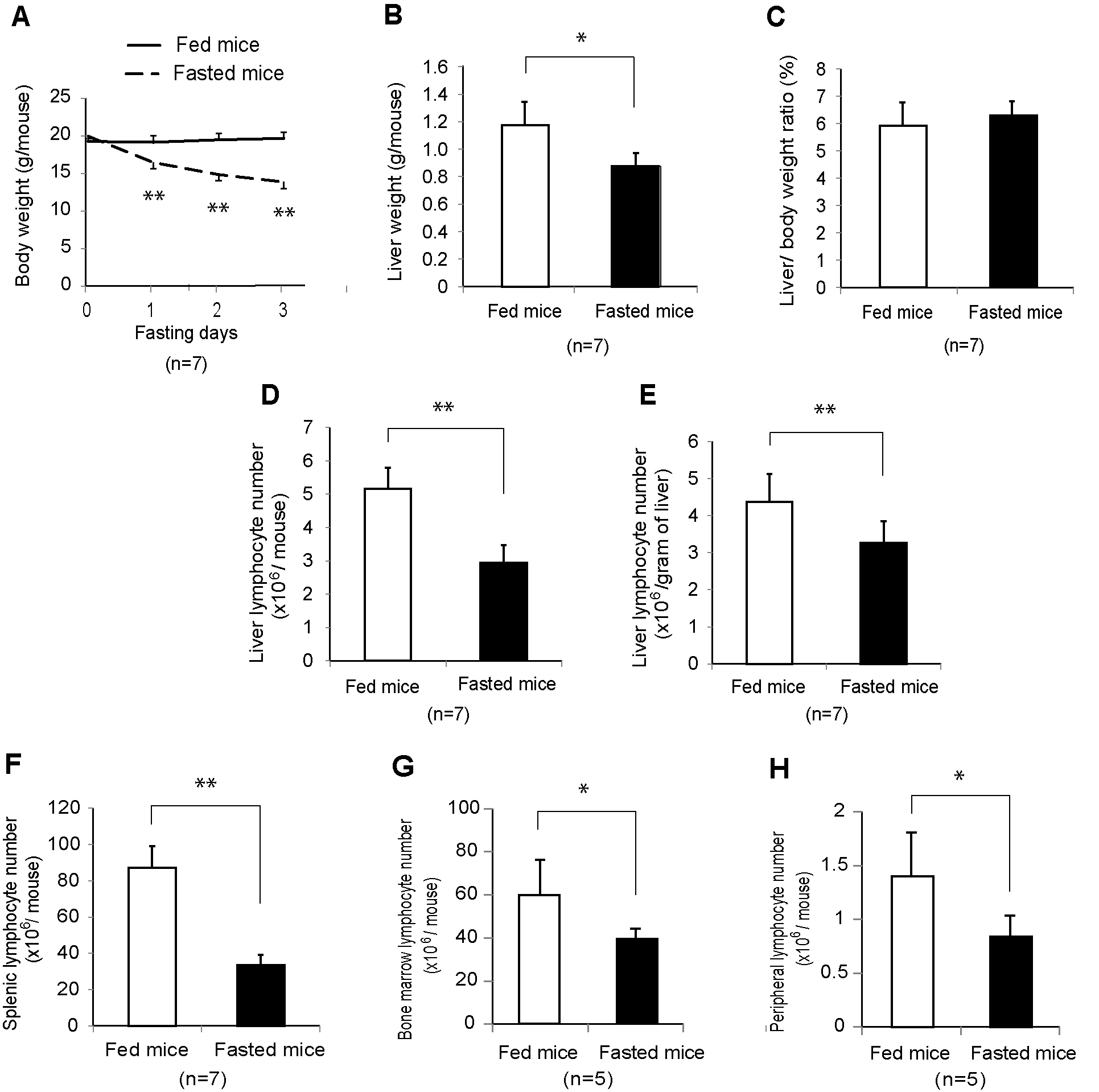

Supplement: Figure S2 — Physiological characteristics of the fasted mice. (A) Mouse body weight was measured every day during the fasting period. (B, C) Liver weight and ratio of liver:body weight were determined on the day of sacrifice. Lymphocytes from (D, E) the liver, (F) spleen, (G) bone marrow, and (H) blood from fed and fasted mice were counted using a hemocytometer; average numbers plus standard deviation are shown. The difference between groups was analyzed using the independent samples T test; *p <0.05; **p <0.01. (TIF) [file pone.0110748.s002.tif]
